# Reviving the view: evidence that macromolecule synthesis fuels bacterial spore germination

**DOI:** 10.1093/femsml/uqac004

**Published:** 2022-04-08

**Authors:** Bing Zhou, Sima Alon, Lei Rao, Lior Sinai, Sigal Ben-Yehuda

**Affiliations:** Department of Microbiology and Molecular Genetics, Institute for Medical Research Israel-Canada, The Hebrew University-Hadassah Medical School, POB 12272, The Hebrew University of Jerusalem, 91120 Jerusalem, Israel; Department of Microbiology and Molecular Genetics, Institute for Medical Research Israel-Canada, The Hebrew University-Hadassah Medical School, POB 12272, The Hebrew University of Jerusalem, 91120 Jerusalem, Israel; Department of Microbiology and Molecular Genetics, Institute for Medical Research Israel-Canada, The Hebrew University-Hadassah Medical School, POB 12272, The Hebrew University of Jerusalem, 91120 Jerusalem, Israel; Department of Microbiology and Molecular Genetics, Institute for Medical Research Israel-Canada, The Hebrew University-Hadassah Medical School, POB 12272, The Hebrew University of Jerusalem, 91120 Jerusalem, Israel; Department of Microbiology and Molecular Genetics, Institute for Medical Research Israel-Canada, The Hebrew University-Hadassah Medical School, POB 12272, The Hebrew University of Jerusalem, 91120 Jerusalem, Israel

**Keywords:** Bacillus subtilis, sporulation, spore germination, spore dormancy, spore revival

## Abstract

The Gram positive bacterium *Bacillus subtilis* and its relatives are capable of forming a durable dormant long-lasting spore. Although spores can remain dormant for years, they possess the remarkable capacity to rapidly resume life and convert into actively growing cells. This cellular transition initiates with a most enigmatic irreversible event, termed germination, lasting only for a few minutes. Germination is typified by a morphological conversion that culminates in loss of spore resilient properties. Yet, the molecular events occurring during this brief critical phase are largely unknown. The current widely accepted view considers germination to occur without the need for any macromolecule synthesis; however, accumulating data from our laboratory and others, highlighted here, provide evidence that both transcription and translation occur during germination and are required for its execution. We further underline numerous overlooked studies, conducted mainly during the 1960s–1970s, reinforcing this notion. We propose to revisit the fascinating process of spore germination and redefine it as a pathway involving macromolecule synthesis. We expect our perspective to shed new light on the awakening process of a variety of spore-forming environmental, commensal, and pathogenic bacteria and possibly be applicable to additional organisms displaying a quiescent life form.

## Teaser

In this review, we synthesize old and current data to evoke that, in contrast to current dogma, bacterial spore germination demands macromolecule synthesis.

## Introduction: Entering and exiting dormancy

Members of the Gram-positive *Bacillus* and *Clostridium* genera respond to starvation by initiating the developmental process of sporulation, resulting in the formation of a highly durable spore, among the most resilient life forms known (Stragier and Losick 1996, Driks 2002). Spores are long-lived dormant cells, exhibiting exceptional resistance to extremes of heat, desiccation, radiation, and toxic chemicals. Due to their remarkable robustness, spore-forming pathogenic bacteria, such as *C. difficile* and *B. anthracis*, are highly resistant to antibacterial treatments and are difficult to eradicate (Driks 2002, Henriques and Moran 2007, Hutchison *et al*. 2014, Paredes-Sabja *et al*. 2014, Wells-Bennik *et al*. 2016). The non-pathogenic ubiquitous soil bacterium *B. subtilis* evolved throughout the years to become a paradigm for the study of spore biology [e.g. (Stragier and Losick 1996, Higgins and Dworkin 2012)], and as such will be the focus of this review.

The process of sporulation is typified by the formation of an asymmetric septum, dividing the progenitor cell into a small forespore compartment, which designates the future spore, and a larger mother-cell portion, which nurtures the maturing spore. Subsequently, the forespore is engulfed by the mother-cell, and a thick layer of peptidoglycan, called the cortex, inner and outer shells of proteinaceous coats, and a glycoprotein crust are deposited around the spore. These shielding layers protect the spore from its surroundings, providing resilience properties (Stragier and Losick 1996, Henriques and Moran 2007, McKenney *et al*. 2010, Higgins and Dworkin 2012). In parallel, the spore chromosome is bounded by small acid soluble proteins, mainly SspA and SspB, defending it from damage during the dormant phase (Setlow 2007). Such tight packaging could potentially modify the DNA to become inaccessible for transcription. Molecular dormancy is further achieved by dehydration of the spore core, mainly through the accumulation of pyridine-2, 6-dicarboxylic acid (DPA) in complex with Ca^2+^ (Moir 2006). Entering dormancy appears to proceed several days, during which the spore is still responsive to extracellular cues. Throughout this time, the spore RNA content is modified, according to spore age and temperature of incubation (Segev *et al*. 2012, Camilleri *et al*. 2019). Along with the RNA pool, the levels of additional cytoplasmic molecules, such as 3-phosphoglyceric acid (3PGA) and ribonucleotides, are altered even 30 days post-sporulation (Ghosh *et al*. 2015).

**Figure 1.**
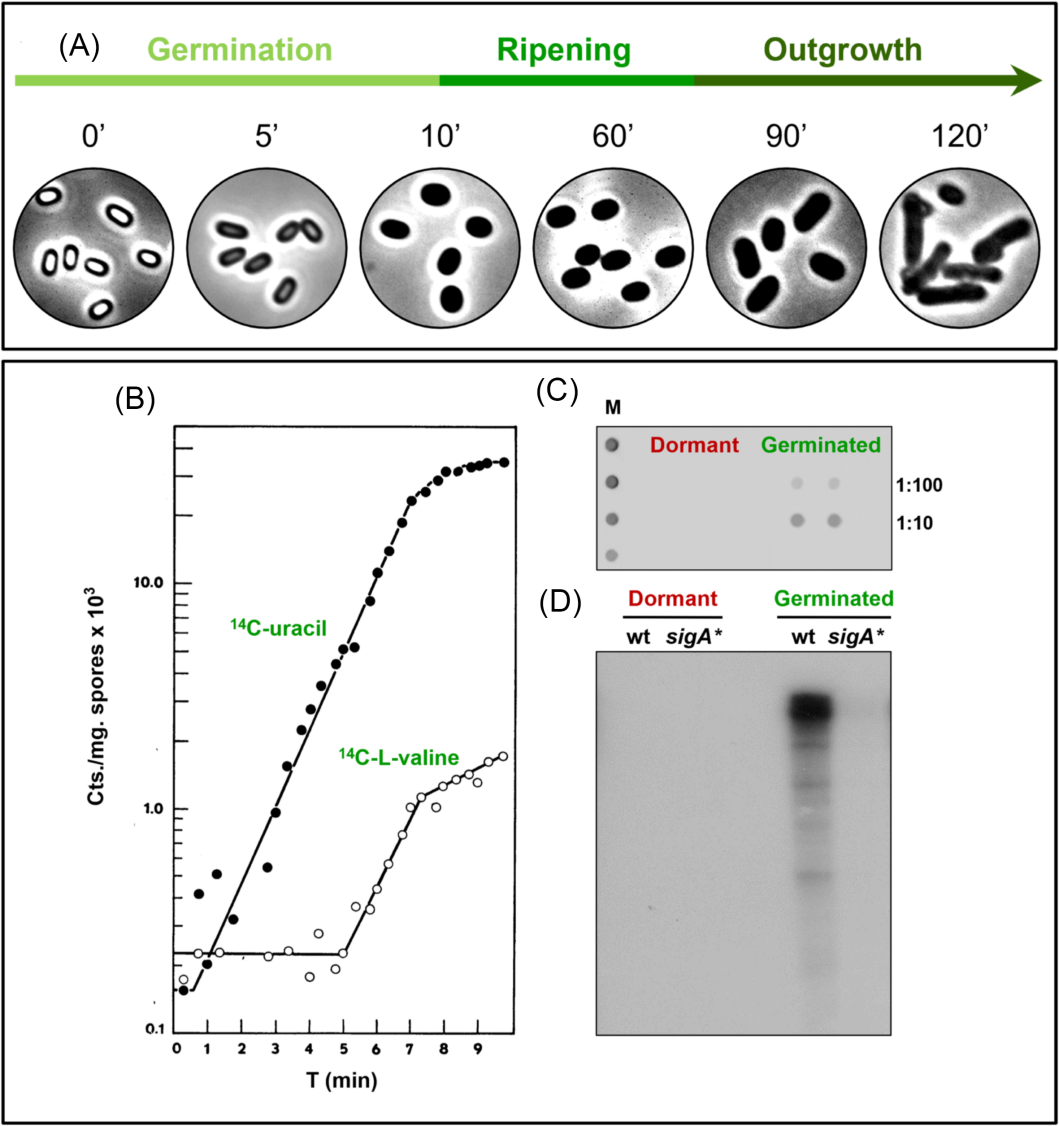
Evidence for the occurrence of transcription and translation during spore germination. **(A)** The morphological sequence of events occurring during spore revival is shown as captured by phase contrast images at the indicated time points [min]. Germination is visualized by a switch from a bright spore to a grey spore and subsequently to a fully germinated dark cell. The ripening period is not associated with an evident morphological change, and outgrowth is characterized by increasing cell length. **(B)** Incorporation of ^14^C-uracil and ^14^C-L-valine during *Bacilli* spore germination provides indication for transcription and translation during germination. Labels were added for clarity. Adopted from (Torriani and Levinthal 1967). Reprinted with permission from the ASM. **(C)** Spores were induced to germinate with the non-nutrient germinant Ca-DPA in the presence of methionine analogue (azidohomoalaine, AHA). Shown is a dot blot analysis of protein samples (in duplicates) that were collected from dormant and germinated spores. Samples were diluted (1:100 and 1:10) and spotted on a membrane that was subsequently probed with anti-biotin antibodies, indicating the presence of AHA. The marker (M) represents known amounts of biotinylated BSA. Adopted from (Sinai *et al*. 2015). **(D)***In vitro* transcription reaction was carried out in whole extracts from dormant and germinated spores of WT and sigA* phospho-mutant strains, in transcription buffer supplemented with NTPs (ATP, CTP, GTP, UTP, [α-32P]-UTP). After 40 min of incubation the reaction was stopped, RNA was purified and radioactively labeled RNAs were analyzed in polyacrylamide gel. Adopted from (Zhou *et al*. 2019).

Spores can remain dormant for years, but still retain the striking potential to promptly resume the vegetative life form once nutrients become reachable (Stragier and Losick 1996, Setlow 2003, Higgins and Dworkin 2012). The process of ***spore revival*** consists of three major consecutive phases: (i) ***Germination***, persisting only for a few minutes, is characterized by a transition from a phase-bright to a phase grey spore, which is then converted to a phase-dark cell (Fig. 1A). During this progression, the spore undergoes rehydration, release of DPA, cortex hydrolysis, and coat disassembly (Setlow 2003, Moir 2006, 2013, Setlow *et al*. 2017); (ii) ***Ripening period***, a lag phase that is dedicated to molecular reorganization that includes massive synthesis of translational and transcriptional components, along with essential metabolic enzymes (Fig. 1A) (Segev *et al*. 2013, Sinai *et al*. 2015); (iii) ***Outgrowth***, in which the spore emerges from the disintegrating shells, and activates the synthesis of macromolecule pathways needed for cell growth and division (Fig. 1A) (Santo and Doi 1974, Sloma and Smith 1979, Setlow 2003, Moir 2006, 2013).

The process of germination, the earliest revival event, is considered most puzzling, and holds the secret to understanding the rapid transition from cellular silencing to cellular awakening. Germination is triggered by the binding of nutrients, termed germinants, to multiple germination receptors (Ger-receptors) located in the spore membrane (Paredes-Sabja *et al*. 2011). The Ger-receptors are assembled into a large germinosome complex, and serve as “aerials” to survey for external germinant factors that include amino acids, sugars, purine nucleosides, DPA, and cell wall derived muropeptides (Setlow 2003, Shah *et al*. 2008, Griffiths *et al*. 2011, Christie and Setlow 2020). In *B. subtilis*, the GerA receptor binds L-alanine, while the GerB and GerK receptors respond jointly to a germinant mixture consisting of asparagine, glucose, fructose, and potassium ions (AGFK) (Setlow 2003, Moir 2006, 2013), Artzi *et al*. 2021. These ligand-receptor interactions activate scarcely explored downstream signaling events, guiding the spore to revive (Setlow 2003, Moir 2006, Dworkin and Shah 2010, Amon *et al*. 2021). The bacterial post-translational modification, arginine (Arg) dephosphorylation, guided by the Arg-phosphatase YwlE, was found to facilitate germination by dephosphorylation of downstream target proteins. Consequently, the absence of the cognate Arg-kinase, McsB, prompts the process (Fuhrmann *et al*. 2009, Elsholz *et al*. 2012, Schmidt *et al*. 2014, Zhou *et al*. 2019, Huang *et al*. 2021, Zhang *et al*. 2021). As we shall discuss here, the Arg-phosphoproteome provides a major clue to elucidate molecular pathways succeeding the activation of the Ger-receptors.

Germination is broadly considered to be completed without the need for macromolecule synthesis, while the subsequent stages of revival are characterized by the massive production of RNA and proteins (Steinberg *et al*. 1965, Vinter 1970, Setlow 2003, 2013, Christie and Setlow 2020). This assessment heavily relies on previous studies, mostly conducted during the 1960s–1970s, showing that *Bacilli* spores can undergo germination in the presence of RNA and protein synthesis inhibitors [e.g. (Steinberg and Halvorson 1968, Vinter 1970)]. Still, several studies performed back then implied that macromolecule synthesis occurs very early in reviving spores, at a time coinciding with germination, and even in media lacking any growth factors [e.g. (Balassa and Contesse 1965, Torriani and Levinthal 1967, Armstrong and Sueoka 1968)]. Nevertheless, the dogma affirming that germination does not require RNA or protein synthesis was almost unanimously accepted [e.g. (Setlow 2003, Moir 2006, 2013)]. In recent years, our laboratory provided evidence indicating that both transcription and translation are activated during germination and required for its completion. We would like to employ this platform to portray our revised view in conjunction with supporting past and current experiments and discuss this conceptual change and its implications.

### Evidence for the occurrence of translation during germination

The fundamental question of whether protein synthesis is a prerequisite for spore germination has been the subject of numerous earlier studies, executed mainly during the 1960s–1970s. A key strategy of those studies was to supplement spores with an array of translation inhibitors and assess germination capacity. Such assays failed to detect an effect on germination, thus leading to the conclusion that germination is independent of protein synthesis [e.g. (Steinberg *et al*. 1965, Vinter 1970)]. Other researchers challenged these interpretations at that time, claiming that the tested compounds could not cross the impermeable spore shells to reach the targeted ribosomes (Tisdale and DeBusk 1972, Sussman and Douthit 1973). Furthermore, these results were inconsistent with several studies demonstrating the incorporation of radioactive amino acids into newly synthesized proteins within a few minutes following germination induction. For example, Torriani and Levinthal could detect ^14^C-L-valine incorporation into proteins 3 to 4 minutes post-stimulation of germination (Torriani and Levinthal 1967) (Fig. 1B). Similarly, Shaw and Armstrong assessed protein synthesis in germinating spores by following ^3^H-phenylalanine incorporation specifically into components composing the 50S ribosomal subunit. Surprisingly, they found that synthesis of the majority of the 50S ribosomal proteins is detectable as early as 5–10 minutes post-germination triggering (Shaw and Armstrong 1972), signifying that translation is likely to initiate even earlier than the detected time, and hence overlaps with the process of germination. In keeping with these findings, polysomes were shown to exist in dormant spores (Chambon *et al*. 1968, Feinsod and Douthit 1970), hinting that they could be rapidly tuned to translate the associated transcripts upon germination. Coupled with this, biochemical studies showed the existence of different-size ribosomal particles from dormant and germinating spores, inferring prompt modulation of ribosomal complexes during the transition from dormancy to germination [e.g. (Chambon *et al*. 1968, Idriss and Halvorson 1969, Feinsod and Douthit 1970)].

In the current years, our lab provided evidence reinforcing the view that translation occurs during germination and is essential for its execution (Sinai *et al*. 2015, Zhou *et al*. 2019). These unexpected findings came as a surprise while employing the BONCAT (BioOrthogonal Non-Canonical Amino-acid Tagging) protein tagging technique to study the temporal landscape of newly synthesized proteins during spore revival (Dieterich *et al*. 2007, Sinai *et al*. 2015). Using this approach, the generation of labeled proteins was monitored when spores were triggered to germinate with L-alanine or with the “non-nutrient” germinant Ca-DPA, in the absence of any other nutrients that are required to support successive revival events (Fig. 1C) (Sinai *et al*. 2015). By treating dormant spores with ethanol to increase permeability (Tanimoto *et al*. 1996), we could introduce into the spore core ribosome-targeting antibiotics that subsequently halted labeled-protein production. Furthermore, the antibiotic-treated spores could initiate germination, as indicated by DPA release, but were paused prior to cortex hydrolysis and thus remained phase bright (Sinai *et al*. 2015). The DPA release suggests that at least partial core rehydration occurred, allowing resumption of protein synthesis among other enzymatic activities. Based on the BONCAT methodology, a set of 30 proteins synthesized during this early stage was defined as the basic germination proteome. The proteome contained proteins required for glycolysis, malate utilization and translation, along with enzymes composing the vital pyruvate dehydrogenase complex (Sinai *et al*. 2015). Notably, included in the germination proteome were two *bona fide* translational factors, RpmE, a ribosomal component constituting the 50S subunit (Akanuma *et al*. 2012), and Tig, a ribosome-associated chaperone (Wegrzyn and Deuerling 2005). Spores lacking these factors were halted in germination due to deficiency in protein synthesis, directly linking these factors with the process of germination-induced translation (Sinai *et al*. 2015). In line with these findings, dephosphorylation of Tig, phosphorylated on the Arg-residue (R45) by YwlE, was found to propel germination, enabling the association of Tig with the ribosomes to reactivate protein synthesis (Zhou *et al*. 2019).

The conceptual shift that translation occurs during germination and is required for its occurrence was received with skepticism by some researchers, opposing this prospect (Korza *et al*. 2016, Christie and Setlow 2020, Swarge *et al*. 2020b). We carefully assessed these studies, and, to our understanding, the illustrated experiments seem indecisive and lack appropriate controls [e.g. (Sinai and Ben-Yehuda 2016)]. For example, the use of antibiotics or the employment of *rpmE* or *tig* translational mutants were not examined by these researchers to sustain or rule out our findings (Boone and Driks 2016). Besides, as we mentioned above, our results are in agreement with numerous earlier deserted studies, reporting that translation can be monitored within a few minutes post-germination triggering (Fig. 1B) [e.g. (Torriani and Levinthal 1967, Rodenberg *et al*. 1968, Steinberg and Halvorson 1968, Shaw and Armstrong 1972)].

### Activation of and the requirement for transcription during germination

The detection of germination-induced translation raises the immediate question as to whether *de novo* transcription is entailed in the process. In fact, numerous early observations showed the occurrence of RNA synthesis in spores almost immediately after prompting germination [e.g. (Torriani and Levinthal 1967, Armstrong and Sueoka 1968, Rodenberg *et al*. 1968, Cohen and Keynan 1970, Matsuda and Kameyama 1986)]. Torriani and Levinthal, for instance, reported that incorporation of ^14^C-uracil into germinating spore RNA occurs as fast as 30–60 sec following germination induction, whereas Amstrong and Sueoka monitored uridine incorporation within two minutes after germinant supplementation (Torriani and Levinthal 1967; Fig. 1B). Armstrong and Sueoka 1968 Co-examination of both RNA and protein synthesis in germinating spores by various studies steadily showed that RNA production precedes protein manufacturing [e.g. (Torriani and Levinthal 1967, Armstrong and Sueoka 1968, Rodenberg *et al*. 1968)], hinting that there is a firm hierarchy in reactivation of these processes. In a recent transcriptomic analysis of reviving spores, Swarge et al (Swarge *et al*. 2020a) concluded that there is no significant change in the spore transcriptome during germination. However, they do detect that at the time coinciding with germination completion, transcripts associated with purine and pyrimidine biosynthesis (14 transcripts), amino acid production (9 transcripts), and translational components (31 transcripts) were upregulated, which seems consistent with our view.

Evidence for the necessity for transcription during germination was unexpectedly gained while exploring the spore Arg-phosphoproteome, through which the occurrence of spore-unique Arg-phosphorylation of the housekeeping sigma factor σ^A^, was uncovered (Haldenwang 1995, Zhou *et al*. 2019). The construction of a mutant strain, mimicking a constitutive phosphorylation state, unveiled the corresponding spores to have a profound deficiency in germination, without any apparent influence on vegetative growth (Zhou *et al*. 2019). Further examination revealed that the mutant spores were stalled before DPA release and remained heat resistant, indicating a deficiency at an early germination stage, prior to protein synthesis activation. Subsequent analysis monitored an increase in transcript levels of genes included in the germination proteome (i.e., *tig* and *rpmE*) in wild type, but not in spores harboring a constitutively phosphorylated *sigA* mutation. An *in vitro* system devised for monitoring the transcriptional activity of whole extracts failed to detect transcription by dormant spore extracts. However, triggering germination was sufficient to reactivate the transcriptional machinery in extracts from wild type spores but was undetectable in extracts of *sigA* phospho-mutant germinating spores (Fig.   1D) (Zhou *et al*. 2019). These findings indicate that the transcription machinery ceases at the course of dormancy, reinitiates early during germination, and is required to advance the process. This rapid molecular awakening is mediated, at least in part, by Arg-dephosphorylation of SigA by YwlE. The massive RNA degradation observed in spores entering dormancy (Segev *et al*. 2012, Segev *et al*. 2013, Camilleri *et al*. 2019) alludes that the resultant ribonucleotides are used as a reservoir to be consumed through germination. We assume that the subsequent awakening of the translational machinery is fueled by the germination-synthesized transcripts that couple with the ribosomes. In the future, examination of these early-produced transcripts might assist in illuminating the process.

### Germination is propelled by phosphorylation and dephosphorylation events

Among the most puzzling germination events is the delivery of a signal from the stimulated germinant receptor into the spore core, conveying the fast resumption of multiple cellular processes including transcription and translation. This primary event is still enigmatic, yet accumulating evidence implies that the co-initiation of various processes is facilitated, at least partially, by protein phosphorylation, a ubiquitous means for mediating rapid cellular responses to external stimuli (Kobir *et al*. 2011). In fact, an assortment of protein kinase inhibitors was found to interfere with spore awakening in the bacterium *Streptomyces coelicolor* (Paleckova *et al*. 2007), whereas muropeptides were shown to trigger spore germination in *B. subtilis* by activating the eukaryotic-like membrane serine/threonine kinase PrkC (Shah *et al*. 2008). In line with these findings, phosphoproteomic analysis, defining the Ser/Thr/Tyr phosphoproteome of dormant and reviving spores, supports the notion that phospho-modifications play a central role in rapidly modulating protein activity throughout revival. However, evidence for the direct impact of these phosphorylation events on the actual germination process has not been assigned (Rosenberg *et al*. 2015). By conducting a genetic unbiased screen to isolate mutants, deficient in germination, we recently identified the arginine phosphatase YwlE as a facilitator of the process (Zhou *et al*. 2019). Arg- phosphorylation, unique to Gram positive bacteria (Elsholz *et al*. 2012, Schmidt *et al*. 2014, Trentini *et al*. 2016), seems to acquire an exclusive role in spore-forming bacteria, controlling spore dormancy and awakening. Besides the role of YwlE in resuming translation and transcription by dephosphorylating Tig and SigA respectively, the spore Arg-phosphoproteome hints that it could also stimulate the awakening of key metabolic pathways. For instance, GapA, glyceraldehyde-3-phosphate dehydrogenase and Pgi, glucose-6-phosphate isomerase, harbor Arg-phosphorylation sites unique to spores (Zhou *et al*. 2019), suggesting that YwlE instantly reactivates a plethora of pathways, propelling the exit from dormancy. Though, how the phosphatase activity is unleashed during germination, how it receives the signal from the germination receptors, and whether arginine phosphorylation is only part of an array of multiple phosphorylation-dephosphorylation events that act to resume life remains unrevealed.

### Spores are not all made equal

Besides obvious genetic and environmental factors that affect spore germination properties, it becomes evident that phenotypic variation in the spore's temporary molecular cargo impacts its ability to germinate. A prominent observation was that a poorly germinating subpopulation exists in a genetically identical spore population. The germination deficiency of these superdormant spores was found to originate from low levels of germination receptor components due to phenotypic variation (Ghosh and Setlow 2009, Ghosh *et al*. 2012, Chen *et al*. 2014). In complementary findings, Sturm and Dworkin showed that spores germinate stochastically due to fluctuating expression of the sporulation transcription factor GerE, involved in the last steps of spore assembly, with spores having the lowest expression of *gerE* showed the highest frequency of spontaneous germination (Sturm and Dworkin 2015). A more recent study that followed the life cycle of individual cells using time-lapse microscopy exposed that sporulation timing controls spore revival, most probably due to variations in the levels of the metabolic enzyme alanine dehydrogenase (Mutlu *et al*. 2018, Mutlu *et al*. 2020). This is consistent with the observations that varying sporulation conditions interferes with germination [reviewed in (Bressuire-Isoard *et al*. 2018)]. For instance, spores formed in nutrient-poor sporulation medium germinated more slowly than spores formed in nutrient-rich medium (Ramirez-Peralta *et al*. 2012). Taken together, these findings reinforce the view that phenotypic diversity during the pre-spore period affects spore germination and revival capabilities. Furthermore, while following the RNA content of mature spores over time, we found that a few days after sporulation, the RNA profile is dynamically modified, depending on the spore age and the temperature of incubation. Consequently, the ability to germinate and propagate varies, implying that molecular changes occurring after the conclusion of sporulation continue to shape the spore molecular pool and influence the emergence from quiescence (Segev *et al*. 2012). As *B. subtilis* and its relatives reside in diverse ecological niches, different characteristics of each habitat are likely to produce spores harboring dissimilar molecular cargo, impacting germination kinetics.

### Perspective

**Figure 2.**
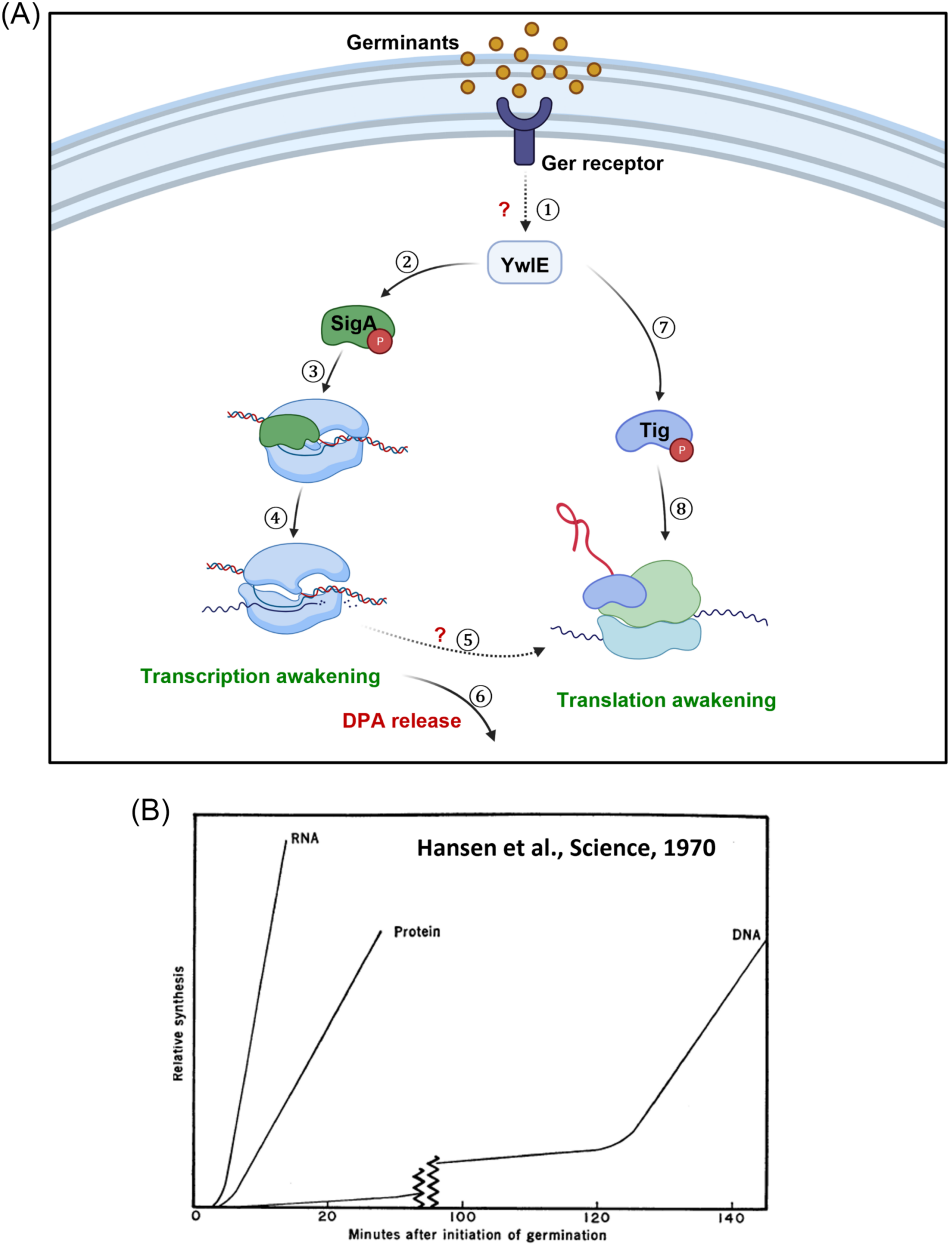
A revised model for bacterial spore germination. **(A)** A revised view for bacterial spore germination. Following nutrient sensing, the germination receptors activate the Arg phosphatase YwlE (①). Consequently, SigA is dephosphorylated by active YwlE to reactivate the transcription machinery (②③④). Next, translation is reestablished by the YwlE-mediated dephosphorylation of Tig after DPA release (⑥⑦⑧). The source of the transcripts for protein synthesis during germination could be derived from the newly transcribed RNA (⑤). **(B)** Our revised model very much resembles that of Hansen et al. (Hansen *et al*. 1970) based on their results. Reprinted with permission from the AAAS.

Dormant bacterial spores can survive prolonged periods and withstand extreme conditions but preserve the extraordinary capacity to switch within minutes into actively growing cells, with germination being the initial and most critical event of this transformable process. As we attempted to illuminate throughout this perspective, we suggest that the notion of germination being independent of macromolecule synthesis needs to be reviewed, as accumulating data concomitantly with previous observations, indicate that both transcription and translation are restored during germination and required for its culmination. Our current viewpoint is that the intricate process of spore germination initiates by activation of Ger-receptors that, in turn, activate downstream signal transduction pathways, with the key path comprising the arginine phosphatase YwlE, which dephosphorylates target proteins implicated in metabolism, transcription, and translation. Dephosphorylation of SigA activates transcription (before DPA release), whereas subsequent dephosphorylation of Tig activates translation (after DPA release) (Fig. 2A). Intriguingly, our model greatly resembles that of Hansen, Spiegelman, and Halvorson proposed based on their data obtained approximately 50 years ago (Fig. 2B; Hansen *et al*. 1970). Our model is also supported by previous studies showing that the spore reservoir contains all elements needed for initial macromolecule synthesis, including nucleotides, and amino acids [e.g. (Kieras *et al*. 1978, Setlow and Kornberg, 1970, Setlow and Primus, 1975)]. Moreover, the spore holds high concentrations of potential energy sources such as PGA and malate that could be employed for raising the ATP levels during germination (Nelson and Kornberg 1970, Ghosh *et al*. 2015, Sinai *et al*. 2015). The revised view we present here might pave the way for developing innovative strategies to interfere with spore germination of dangerous pathogens and those causing food spoilage. It could further enlighten the awakening process of various bacteria exhibiting a latent phase, and be applicable to diverse organisms displaying a dormant form, including fungi spores and plant seeds.

## Funding

This work was supported by the Israel Science Foundation (grant no. 774/16, 308/21) awarded to S. B-Y.
